# Autoimmune polyglandular syndrome type 1 with diabetes insipidus: a case report

**DOI:** 10.1186/s12902-021-00822-6

**Published:** 2021-08-03

**Authors:** JiaQi Chen, Ting Lu, ChenXiao Liu, Yun Zhao, AiJie Huang, XingNa Hu, Min Li, Rong Xiang, Min Feng, HongHong Lu

**Affiliations:** Department of Endocrinology and Metabolism, The Affiliated Suzhou Hospital of Nanjing Medical University, Suzhou Municipal Hospital, Suzhou, Jiangsu China

**Keywords:** Autoimmune polyendocrine syndrome type 1, Diabetes insipidus, Autoimmune regulator gene

## Abstract

**Background:**

Autoimmune polyendocrine syndrome type 1 (APS-1) is a rare monogenic inherited disease caused by mutations of the autoimmune regulator gene (AIRE). The three major components of this syndrome are chronic mucocutaneous candidiasis, hypoparathyroidism and adrenocortical insufficiency.

**Case presentation:**

We report a 20-year-old male who was clinically diagnosed with APS-1 at the age of 15. He was admitted to our department this time for suffering from polyuria and polydipsia for 6 months and was finally diagnosed with diabetes insipidus. Whole-exome sequencing (WES) revealed a novel compound heterozygous mutation of the AIRE gene —the c.239 T > G (p.Val80Gly) variant on one allele and the copy number variant (CNV) of 21q22.3(chr21:45,670,150–45,706,528)*1 on the other.

**Conclusions:**

This case suggests that diabetes insipidus is a rare component of APS-1 and expands the variety of mutations on AIRE gene.

## Background

Autoimmune polyendocrine syndrome type 1 (APS-1) is a rare monogenic inherited disease caused by mutations of the autoimmune regulator gene (AIRE) located on chromosome 21q22.3 [1]. The prevalence of APS-1 is quite low and varies in different populations. It is relatively higher in Iranian Jews (1:9000), Sardinians (1:14,400), and Finns (1:25,000) [2–4]. However, APS-1 is quite rare in Eastern Asians [5, 6].

The classical triad of this syndrome is chronic mucocutaneous candidiasis, hypoparathyroidism, and adrenal insufficiency. The clinical diagnosis is based on the presence of at least two of the three symptoms. As AIRE has a crucial role in promoting self-tolerance in the thymus, APS-1 patients may also present other autoimmune disorders such as autoimmune thyroiditis, type 1 diabetes, hypertrophic hypogonadism, and pernicious anemia [7]. As the clinical manifestations varies, the genetic and immunological features are also important in the diagnosis of this syndrome [8]. In this manuscript, we reported a Chinese APS-1 patient with a rare component— central diabetes insipidus. The genetic analysis revealed a novel compound heterozygous mutation.

## Case presentation

A 20-year-old Chinese male was admitted to our department due to the complaint of polyuria and polydipsia for six months. The patient was born of non-consanguineous parents and was clinically diagnosed with APS-1 5 years ago. The patient suffered from chronic mucocutaneous candidiasis since the age of 9 years. Both his nails and oral cavity were affected. Later on he developed adrenal insufficiency and autoimmune thyroiditis at the age 15 years. Alopecia was also presented when he was 15 years old. The patient was then clinically diagnosed as APS-1 by us and visited our department once a year for regular follow-ups. The adrenal and thyroid function were evaluated every follow-up and the dose of hydrocortisone and levothyroxine were adjusted accordingly. The serum calcium level and PTH level were also screened every year and no abnormal results were found. There were no other important complaints in the 5-year-follow-up until he was 20 years old. The symptoms and managements are summarized in Table 1.

**Table 1:** The presentations of the patient

Components	Age of diagnosis	Presentation	Treatment
Chronic mucocutaneous candidiasis	9	Oral cavity and nails candidiasis	Course of antifungal drugs
Adrenal insufficiency	15	Hyperpigmentation, weakness and fatigue	Maintenance therapy (hydrocortisone)
Autoimmune thyroiditis	15	Subclinical hypothyroidism and high titers of antibodies	Levothyroxine
Alopecia	15	Hair loss	No treatment
Diabetes insipidus	20	polyuria and polydipsia	Desmopressin

**Fig. 1:**
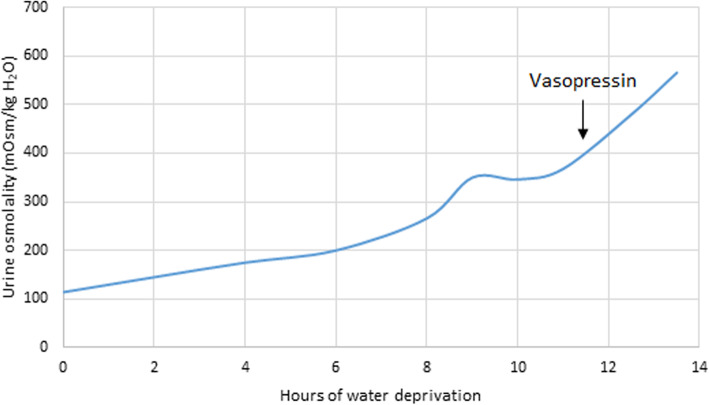
Urine osmolality during a dehydration test. Vasopressin was administrated 11.5 h after water deprivation

As being a common component of APS-1, type 1 diabetes was our first consideration. However, oral glucose tolerance test revealed that fasting and 2-h glucose levels were 4.25 mmol/L and 4.39 mmol/L, respectively. The HbA1c level was 4.96%. Thus, diabetes mellitus was excluded. Meanwhile, adrenal function, thyroid function, and parathyroid function were reevaluated. All the results were within normal range. Interestingly, the 24 h urine output of the patient was 9.1 L. And the patient’s urine test revealed that urine specific gravity was 1.004 and osmolality was 114 mOsm/kg H2O. Impression of diabetes insipidus was highly suspected and water deprivation test and vasopressin administration was then performed. The result (Fig. 1) suggested that our patient suffered from central diabetes insipidus. Urine osmolality was elevated from 114 mOsm/kg H2O to 368 mOsm/kg H2O during water deprivation and was further increased to 566 mOsm/kg H2O after vasopressin administration. And plasma osmolality before water deprivation test and before and after vasopressin administration were 300, 313, and 314 mOsm/kg H2O, respectively. A further MRI was performed and there were no significant findings in pituitary and hypothalamus. Nor were tumors observed in MRI imaging. Desmopressin of 0.1 mg every 8 h was then prescribed to the patient and the symptoms improved. Urine output was approximately 3 L per day when the patient was discharged.

**Fig. 2:**
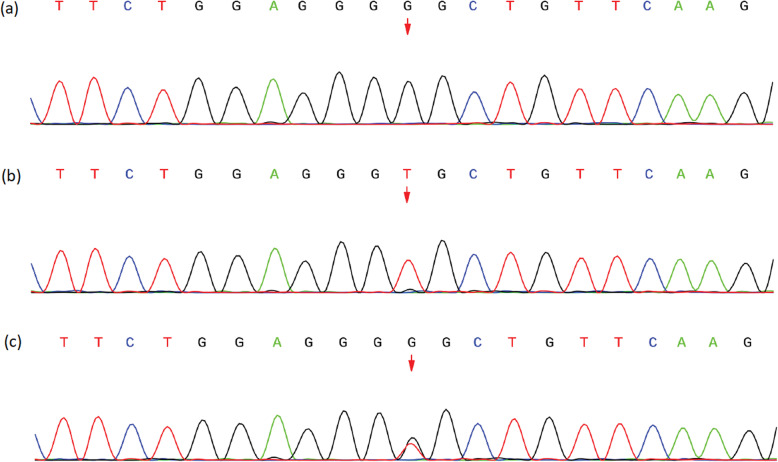
Sanger sequencing chromatogram of AIRE gene. **a** The patient carries the variant of c.239 T > G (p.Val80Gly). **b** The patient’s father is a wildtype. **c** The patient’s mother carries the same heterozygotes variant

Furthermore, Whole-exome sequencing (WES) was performed this time. WES revealed a compound heterozygous mutation of the AIRE gene —the c.239 T > G (p.Val80Gly) variant on one allele and the copy number variant (CNV) of 21q22.3(chr21:45,670,150–45,706,528)*1 on the other. Both patient’s parents were examined for this mutation. The patient’s mother carries the same heterozygotes variant of c.239 T > G (p.Val80Gly), while the father is a wildtype (Fig. 2).

## Discussion and conclusion

In our case, we described a new novel compound heterozygous mutation of the AIRE gene in this patient clinically diagnosed as APS-1. The c.239 T > G (p.Val80Gly) variant was previously reported [9], while the CNV of 21q22.3 (chr21:45,670,150–45,706,528)*1 is newly discovered.

As mentioned above, AIRE promotes the expression of tissue specific antigens in medullary thymic epithelial cells for negative selection of autoreactive T effector cells [7]. Up to date, more than 100 mutations have been reported in AIRE, varying from single nucleotide variant to large deletions across exons. Most mutations are autosomal-recessively inherited. As some certain populations have higher prevalence of this disease, several mutations are more common among these populations [10, 11]. The p.Y85C is more common in Iranian Jewish APS-1 patients. The p.R139X mutation is the most common in Sardinia patients and seems to be related with a more severe clinical phenotype. The p.R257X is a typical mutation in Finnish and Russian patients. The c.967-979del13bp mutation is also common in different populations.

AIRE consists of four major functional domains: the CARD domain, the SAND domain, and two PHD domains. The CARD domain is transcripted and translated from exon 1 and 2, which was affected in our case. The CARD domain is involved in the process of AIRE homomultimerization, which is essential to perform its function [12, 13]. It was believed that AIRE formed dimerization or tetramerization to play its function. However, a recent research found that it was vital for its function that AIRE CARD domain forms filaments and large assembly rather than the formation of dimerization or tetramerizaiton [14]. And furthermore, this assembly mediates foci formation and transcriptional activity of AIRE [14].

In our case, both of the two variants locate on CARD domain. The variant of c.239 T > G(p.Val80Gly) was previously reported in two Indian patients [9]. This mutation was believed to be pathogenic while it is homozygous. Both of the two patients had this homozygous mutation. One of the patients was the product of a consanguineous marriage. As for the other patient, both her parents carried a heterozygotes variant. And she had an unaffected brother. In our case, the mother of our patient who also carried this heterozygotes variant was not affected. The CNV of 21q22.3(chr21:45,670,150–45,706,528)*1 including AIRE exon 1 and 2 makes one allele of AIRE short of CARD domain. Both of our patient’s parents were not found to carry this CNV. We think the CNV itself may affect the function of AIRE as a transcriptional regulator. However, we believe that, in our case, the CNV makes the c.239 T > G (which locates on AIRE exon 2) variant on the other allele to some extent “homozygous”, and the compound heterozygous mutation led to the development of APS-1.

Although APS-1 is a monogenic disease, there is no strong correlation between genotype and clinical phenotype. A wide spectrum of clinical manifestations have been reported in APS-1 cases. And clinical expressions may differ even in siblings carrying the same genetic variant. However, some researchers proposed a clinical classification of APS-1, which to some extent, associates with genetic variant types: the “classical” and “non-classical” APS-1 [15]. The “classical” APS-1 is characterized by the presence of at least two of three main components and autoimmune antibodies, and is homozygous or compound heterozygous recessive inherited. The “non-classical” APS-1 varies in clinical phenotype from the main components of APS-1 to isolated organ-specific autoimmunity and is usually inherited in a heterozygous dominant manner. Our patient suffered from chronic mucocutaneous candidiasis and adrenal insufficiency. These two clinical features makes the clinical diagnosis of APS-1. Together with the compound heterozygous mutation, our patient met the criteria of “classical” APS-1.

Reported by previous studies [2, 4, 16–18], chronic mucocutaneous candidiasis is the most common and usually the first component of APS-1, and it usually occurs before the age of 5. Hypoparathyroidism is the most common endocrine component and usually appears after chronic mucocutaneous candidiasis. Adrenal insufficiency is less common than hypoparathyroidism and has an incidence peak at the age of 12 years. The most three frequent minor manifestations are autoimmune thyroiditis, type 1 diabetes, and autoimmune hepatitis. Our patient developed chronic mucocutaneous candidiasis at the age of 9 years and then developed adrenal insufficiency and autoimmune thyroiditis at the age 15 years. He also presented alopecia, which is also a common minor components of APS-1 reported in a Russian cohort with 112 APS-1 patients [11].

Interestingly, our patient was referred to us for his polyuria and polydipsia. Water deprivation test and vasopressin administration revealed that our patient suffered from central diabetes insipidus. Further pituitary MRI found no significant lesions. The causes of central diabetes insipidus are usually pituitary lesions, including trauma, tumor, or infection. Lymphocytic filtration is now recognized also a common cause of central diabetes insipidus. And it may recover to normal while diabetes insipidus is left permanent. There are only few cases of diabetes insipidus caused by APS. In a study [19] including 79 children and young adults with central diabetes insipidus, only in one patient diabetes insipidus was caused by APS. And the patient developed polyuria and polydipsia until age 24.8 while he had been diagnosed with APS at the age of 2 years. The long period of time elapsed since APS was diagnosed to the presentation of diabetes insipidus is also seen in our patient. Another cohort [20] from Turkey including 34 children with central diabetes insipidus revealed that only in one case diabetes insipidus was caused by APS. The development of central diabetes insipidus in APS patients may be due to autoimmune process. In a large cohort of 2385 APS patients, 22 of them with central diabetes insipidus, and most of them developed diabetes insipidus after the age of 20 years, was selected out for screening for antibodies to AVP-secreting cells (AVPcAb). AVPcAb was positive in 15 of the 22 patients and consisted positive during the 5-year follow-up. While in the control group of 13 idiopathic central diabetes insipidus, only 5 of them were AVPcAb positive [21]. The presence of AVPcAb suggested that due to the autoimmune pathogenesis, diabetes insipidus is also a component in APS. The damage of AVP cells may be chronic, which may be associated with the late development of central diabetes insipidus in APS patients.

Through this case, we reported a novel compound heterozygous mutation of AIRE in an APS-1 patient. More importantly, we discussed the clinical components of APS-1 and learned that central diabetes insipidus may also be an important component of APS-1. For patients, especially children and young adults, with “idiopathic” central diabetes insipidus and other endocrine deficiencies, APS-1 must be considered. Genetic analysis and AVPcAb should be performed if the tests are available.

## Data Availability

All data related to this report are stored at Nanjing Medical University affiliated Suzhou Hospital (Jiangsu Province, China), and are available from the corresponding author on reasonable request.

## Abbreviations

AIRE: Autoimmune Regulator gene
APS-1: Autoimmune Polyglandular Syndrome type 1
AVPcAb: Antibodies to AVP-secreting cells
CNV: Copy number variant
WES: Whole-exome sequencing
